# Specification of *Drosophila* Corpora Cardiaca Neuroendocrine Cells from Mesoderm Is Regulated by Notch Signaling

**DOI:** 10.1371/journal.pgen.1002241

**Published:** 2011-08-25

**Authors:** Sangbin Park, Erika L. Bustamante, Julie Antonova, Graeme W. McLean, Seung K. Kim

**Affiliations:** 1 Department of Developmental Biology, Stanford University School of Medicine, Stanford, California, United States of America; 2 Howard Hughes Medical Institute, Stanford, California, United States of America; 3 Department of Medicine (Oncology), Stanford University School of Medicine, Stanford, California, United States of America; Harvard Medical School, Howard Hughes Medical Institute, United States of America

## Abstract

*Drosophila* neuroendocrine cells comprising the corpora cardiaca (CC) are essential for systemic glucose regulation and represent functional orthologues of vertebrate pancreatic α-cells. Although *Drosophila* CC cells have been regarded as developmental orthologues of pituitary gland, the genetic regulation of CC development is poorly understood. From a genetic screen, we identified multiple novel regulators of CC development, including Notch signaling factors. Our studies demonstrate that the disruption of Notch signaling can lead to the expansion of CC cells. Live imaging demonstrates localized emergence of extra precursor cells as the basis of CC expansion in *Notch* mutants. Contrary to a recent report, we unexpectedly found that CC cells originate from head mesoderm. We show that Tinman expression in head mesoderm is regulated by Notch signaling and that the combination of Daughterless and Tinman is sufficient for ectopic CC specification in mesoderm. Understanding the cellular, genetic, signaling, and transcriptional basis of CC cell specification and expansion should accelerate discovery of molecular mechanisms regulating ontogeny of organs that control metabolism.

## Author Summary

The requirement for glucose regulation is conserved in metazoans and crucial for metabolism, growth, and survival. In fruit flies and other insects, neurons secrete insulin-like hormones and neuroendocrine corpora cardiaca cells secrete adipokinetic hormone, a peptide with functional similarities to glucagon. Both hormones are essential for systemic glucose control in *Drosophila*. To understand the mechanisms governing formation and function of corpora cardiaca cells, we sought to identify their embryonic origin and investigate their developmental genetic regulation. Based on prior reports suggesting a neuroectodermal origin, we were surprised to discover—using genetic lineage tracing methods—that embryonic corpora cardiac progenitors derive from anterior head mesoderm. To our knowledge, this is the first demonstration of neuroendocrine differentiation from mesoderm in *Drosophila*. Genetic studies reveal that Notch signaling restricts the number of corpora cardiaca progenitors, and we show that Notch signaling inactivation results in significant expansion of corpora cardiac cells. Loss- and gain-of-function studies identified transcription factors both necessary and sufficient for corpora cardiaca development. These and other findings reveal similarities in the development of fly corpora cardiaca cells and mammalian neuroendocrine cells that develop in the pancreas, pituitary, and from neural crest.

## Introduction

Recent work has revealed multiple features of evolutionary conservation in endocrine regulation of glucose metabolism. For example, in the fruit fly *Drosophila melanogaster*, insulin-producing cells (IPCs) in the brain and adipokinetic hormone-producing corpora cardiaca (CC) cells in the neuroendocrine ring gland are the respective functional orthologues of mammalian pancreatic β-cells and α-cells [1]–[4]. Insect CC cells resemble neurons in multiple ways; CC cells are peptidergic secretory cells [5] that harbor dense core vesicles [6], and have axon-like projections to vascular, gut and brain targets [3], [4], [7]. Similar to pancreatic islet cells and neuronal cell subsets, CC cells also use K_ATP_ channels to regulate AKH secretion [3]. Targeted CC ablation results in marked hypoglycemia [3], [4], demonstrating their role in glucose homeostasis. Thus, the molecular and physiological mechanisms governing CC endocrine function are strikingly similar to those of vertebrate pancreatic islets and neuroendocrine cells.

Despite their crucial role in regulating systemic glucose balance, the embryonic origin of CC cells remains unclear. Based, in part, on their emergence near embryonic foregut, CC cells were initially proposed to originate from a placode in the foregut that produces the stomatogastric nervous system [8]. The CC cell anlage was later inferred to be the most anterior part of mesoderm, based on studies of gene expression in the embryonic head region [9], [10]. Most recently, it was proposed that the CC cells originate from neuroectoderm-derived neuroblasts [11]. This latest study concluded that CC precursors originate from the same placode in which insulin producing neurons are born, and suggested that the developmental relationship between IPC and CC cells may be similar to that of hypothalamus and neuronal pituitary gland. Likewise, while a survey of candidate mutations revealed several genes required for CC development based on ontogenic similarities to pituitary development [9], a systematic, unbiased mutant screen to identify genetic regulators of CC development has not been previously reported.

Here we used genetic screens and gain-of-function studies to investigate specification of CC cell lineage. From a genetic deficiency screen, we discovered that Notch signaling factors are essential regulators of CC development. Our studies demonstrate that Notch signaling controls the number of emerging CC precursor cells. We unexpectedly found that CC cells develop from head mesoderm. Expression of *tinman* in head mesoderm is regulated by Notch signaling and the combination of *tinman* and *daughterless* is sufficient to specify programs leading to ectopic development of CC cell precursors and their AKH^+^ progeny. Thus our studies reveal genetic and cellular mechanisms underlying precursor specification and expansion of neuroendocrine cells crucial for metabolic homeostasis in *Drosophila*.

## Results

### A deficiency screen identifies novel regulators of corpora cardiaca development

To identify regulators of corpora cardiaca development, we screened 292 lines from the DrosDel deficiency collection [12], corresponding to approximately 50% of the genome. We generated strains harboring the *akh*-RedHStinger (*akh*-RHS) reporter gene which marks the nuclei of CC cells at embryonic stage 17 (see Materials and Methods). We observed that *akh*-RHS^+^ cells were undetectable in 39 deficiency lines, and successfully identified mutations in 18 lines that mapped to 14 genes using publicly available mutant alleles (Table S1). In agreement with the previous study [9], we found that mutations in *giant* (*gt*), *short gastrulation* (*sog*), *sine oculis* (*so*), and *glass* (*gl*) prevented embryonic development of *akh*-RHS^+^ cells. These findings validated our strategy to screen the DrosDel deficiency collection. In addition, we discovered that mutations in *crooked neck (crn)*, *spitz (spi)*, *dimmed (dimm)*, *phyllopod (phyl)*, *double parked (dup)*, *three rows (thr)*, *Polycomblike (Pcl)*, *ETS-domain lacking (edl)*, and *heartless (htl)* also result in the complete loss of AKH-expressing cells (Table S1). Thus, our deficiency line screen has revealed new regulators required for CC development.

### Corpora cardiaca cell expansion from Notch signaling disruption

**Figure 1:**
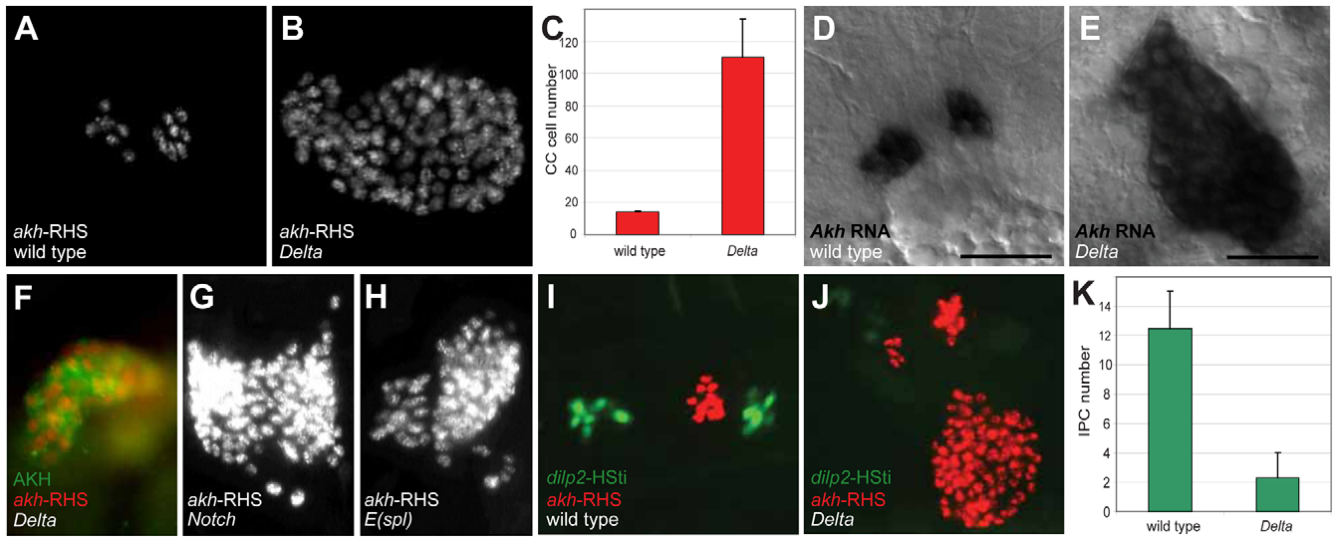
Disruption of Notch signaling results in the expansion of CC cells. (A) Late stage 17 wild type embryo showing 14 CC cells marked by *akh*-RHS. (B) Late stage 17 *Delta* embryo with 93 CC cells marked by *akh*-RHS. (C) Quantification of CC cells in wild type and *Delta* mutants. Average CC cells in wild type embryos is 14.0±0.8 (*n* = 16) while *Delta* mutants show 110.2±23.7 (*n* = 16). (D) *Akh* mRNA *in situ* hybridization in stage 17 wild type embryo. Scale bar is equal to 10 µm. (E) *Akh* mRNA *in situ* in stage 17 *Delta* mutant embryo shows expanded CC cells. (F) Stage 17 *Delta* mutant showing expanded CC cells marked by AKH antibody staining (green) and *akh*-RHS reporter (red). (G–H) Both *Notch* (G) and *E(spl)* (H) mutants at stage 17 show CC cell expansion. (I) Stage 17 wild type embryo show 12 IPCs marked by *dilp2*-HSti reporter (green). (J) Stage 17 *Delta* mutant embryo exhibits reduced IPCs to 4 cells (pale green). (K) Quantification of IPCs in wild type and *Delta* mutants. Average IPCs in wild type embryos is 12.5±2.5 (*n* = 16) while *Delta* mutants show 2.3±1.7 (*n* = 16). Where indicated, data represent the mean ± standard deviation. See also Figure S1.

In contrast to loss of *akh*-RHS^+^ cells in 39 deficiency lines, analysis revealed expansion of *akh*-RHS^+^ cells in the *Df(3R)ED5942* line. The deficiency in this line included the *Delta* gene, which encodes an essential conserved activator of Notch signaling. We subsequently confirmed that *Delta* mutations resulted in the CC cell expansion phenotype observed in *Df(3R)ED5942*. We detected an average of 14.0±0.8 *akh*-RHS^+^ cells in stage 17 control embryos (*n* = 16; Figure 1A and 1C), while in *Delta* mutants we detected an average of 110.2±23.7 *akh*-RHS^+^ cells (*n* = 16; Figure 1B and 1C). *In situ* hybridization and immunostaining revealed expansion of cells expressing *akh* mRNA (Figure 1E) and AKH protein (Figure 1F) in *Delta* mutants, demonstrating expanded CC cells in these mutants. Thus, *Delta* is required for regulating CC cell number.

To identify additional conserved Notch signaling factors required for CC development, we examined *akh*-RHS reporter expression in *Notch*, *Enhancer of split* (*E(spl)*), *Serrate* (*Ser*), and *Suppressor of Hairless* (*Su(H)*) mutant embryos. *Notch* (Figure 1G) and *E(spl)* (Figure 1H) mutant embryos had CC cell expansion indistinguishable from that in *Delta* mutants, while *Ser* and *Su(H)* mutants had no detectable change in CC cell number (data not shown). Together, these findings suggest that Notch signaling restrains development of *Drosophila* CC cells.

A prior study suggested that precursors of CC cells and *Drosophila* insulin producing cells (IPCs) are adjacent in anterior neuroectoderm [11]. To assess the effect of mutations disrupting Notch signaling on IPC development, we generated a *dilp2*-HStinger reporter (*dilp2*-HSti; see Materials and Methods) to mark IPC nuclei and facilitate IPC counting in stage 17 embryos. We detected an average of 12.5±2.5 IPCs (*n* = 16; Figure 1I and 1K) in control embryos, which was significantly different from the average of 2.3±1.7 IPCs in *Delta* mutants (*n* = 16; Figure 1J and 1K). Thus, *Delta* mutants have CC cell expansion accompanied by IPC hypoplasia, and these distinct outcomes suggest that Notch signaling has distinct roles in regulating developmental programs of CC cells and IPCs.

### *Delta* is required before embryonic stage 11 to restrain corpora cardiaca development

**Figure 2:**
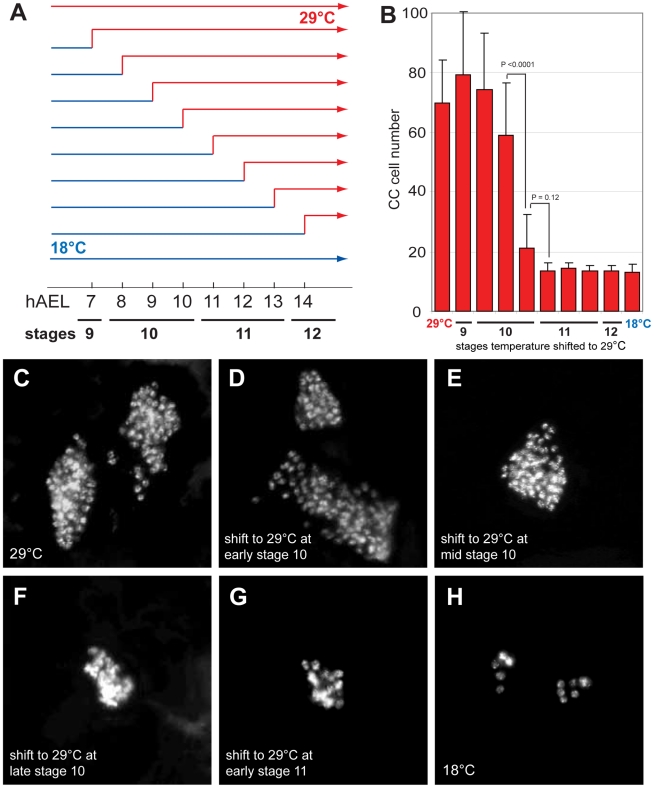
*Delta* regulates CC cell number before embryonic stage 11. (A) Temperature shift conditions applied to *Delta* ^RF^ mutants at different time points following 1-hour egg lay. hAEL is hours after egg lay. (B) CC cell number quantification in *Delta* ^RF^ mutants resulting from the different temperature shift conditions shown in (A). (C) *Delta* ^RF^ mutant grown at 29°C exhibits *akh*-RHS^+^ CC cell expansion. (D) *Delta* ^RF^ mutant grown at 18°C for 8 hours followed by a shift to 29°C until stage 17 shows a similar CC cell expansion. (E) *Delta* ^RF^ mutant shifted from 18 to 29°C at 9 hAEL showing moderate CC cell expansion. (F) *Delta* ^RF^ mutant with a temperature shift at 10 hAEL shows a slight increase in CC cell number. (G) *Delta* ^RF^ mutant with a temperature shift at 11 hAEL exhibits normal CC cell number. (H) *Delta* ^RF^ mutant grown at 18°C shows normal CC cell number. Error bars are ± the standard deviation of the mean.

To determine when *Delta* function is required to restrict CC cell number, we inactivated *Delta* function at specific embryonic stages using the temperature sensitive *Delta* ^RF^ allele. During continuous development at 18°C, CC cell number was normal in *Delta* ^RF^ mutants (13.3±3.1, *n* = 5; Figure 2B and 2H). However, during development at 29°C, CC cell number quadrupled in *Delta* ^RF^ embryos (67.2±27.2, *n* = 13; Figure 2B and 2C), indicating that *Delta* function was efficiently inactivated at 29°C. Based on these findings, we next used temperature shift from 18°C to 29°C at specific developmental stages in *Delta* ^RF^ embryos (summarized in Figure 2A). *Delta* inactivation at 7 or 8 hours after egg lay (hAEL) resulted in CC cell expansion (79.3±20.9, *n* = 11 and 74.3±19, *n* = 30, respectively; Figure 2D). By contrast, lesser CC cell expansion resulted from temperature shift to 29°C at 9 hAEL (59.2±17.6, *n* = 18; Figure 2E) or 10 hAEL (21.3±11.1, *n* = 7; Figure 2F). Shift from 18°C to 29°C at 11 hAEL (corresponding to embryonic stage 11) or thereafter produced CC cell numbers indistinguishable from those observed during continuous development at 18°C (Figure 2B and 2G). These results suggest that *Delta* function is essential for restricting CC cell number before stage 11. To better define better the period when *Delta* restricts CC cell number, we also performed temperature ‘down-shift’ studies at specific stages during *Delta* ^RF^ embryonic development. When the temperature was shifted down from 29°C to 18°C at stage 10, CC cells were not expanded, although their position appeared to be more anterior (Figure S1E). However, temperature shift to 18°C at early stage 11 or thereafter led to CC cell expansion (Figure S1F–S1H). Together, our up- and down-shift experiments suggest that *Delta* is required in a brief period from the end of embryonic stage 10 to the beginning of stage 11 to regulate CC cell number, but may be dispensable before or after.

### The emergence of multiple Glass^+^ CC precursors in *Notch* mutants

**Figure 3:**
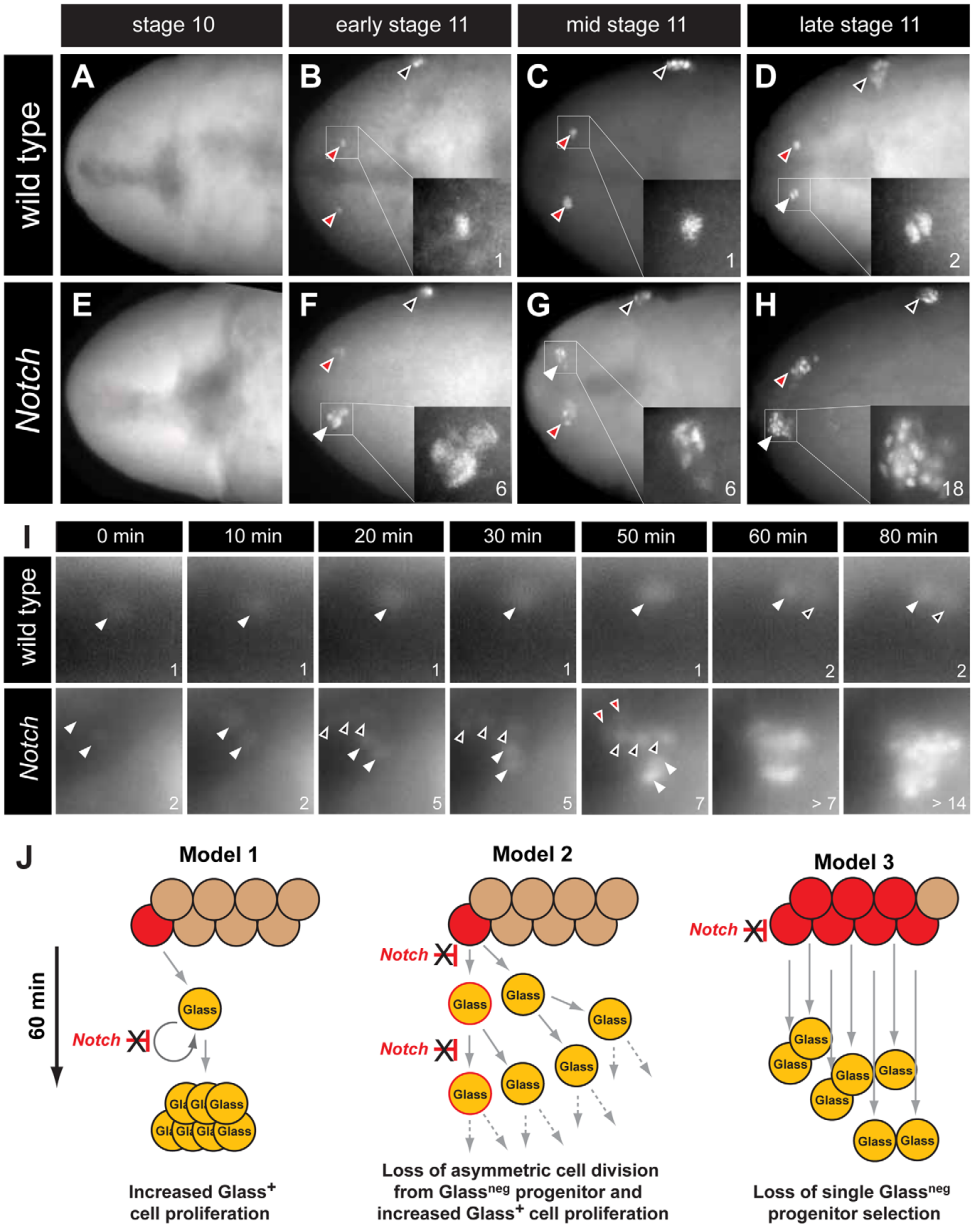
The emergence of multiple Glass^+^ CC precursors in *Notch* mutants. (A–D) CC precursor development in wild type embryo during embryonic stages 10 and 11. Early, mid, and late embryonic stages 11 are determined by Bolwig's organ precursor number (black arrowheads, Material and Methods). (A) Glass expression is not detected in stage 10 wild type embryo. Glass^+^ CC precursors increase from single cell in early-mid stage 11 embryo (red arrowheads and inserts in B and C) to 2 cells in late stage 11 embryo (white arrowhead and insert in D). (E–H) Glass^+^ CC precursors are expanded in *Notch* mutant embryos during embryonic stage 11. (E) Glass expression is not detected in stage 10 *Notch* mutant embryo. (F) *Notch* mutant at early stage 11 showing 2 (red arrowhead) or 6 (white arrowhead and insert) Glass^+^ CC precursors. (G) *Notch* mutant at mid stage 11 showing 4 (red arrowhead) or 6 (white arrowhead and insert) Glass^+^ CC precursors. (H) *Notch* mutant at late stage 11 showing 18 (white arrowhead and insert) Glass^+^ CC precursors. All embryos are dorsal views with anterior to the left. (I) Glass^+^ CC precursors in live wild type and *Notch* mutant embryos are identified by *glass*5.2-RHS expression. The time at which the first precursor is detected is set to 0 minutes. The number of cells counted is labeled in the lower right corner of each image. In wild type, the first CC precursor (white arrowhead) divides at 60 minutes as shown by the emergence of the second precursor (black arrowhead). In *Notch* mutants, two CC precursors first emerge (white arrowheads) at 0 minutes. After 20 minutes, three precursors (back arrowheads) appear without apparent cell division. Two additional precursors (red arrowheads) arise at 50 minutes. The older precursors begin to divide at 60 minutes, indicating that in both the wild type and *Notch* mutants the rate of CC precursor cell division is 60 minutes/division (See also Videos S1 and S2). (J) Models for Glass^+^ CC precursor expansion in *Notch* mutants. Model 1 depicts the possibility that Notch signaling regulates proliferation of Glass^+^ CC precursors. In *Notch* mutants, the speed of this proliferation is increased, resulting in appearance of multiple Glass^+^ CC precursors. Model 2 depicts that Notch signaling normally regulates asymmetric cell division of a Glass^neg^ CC progenitor (red), resulting in one Glass^neg^ progenitor and one Glass^+^ CC precursor followed by subsequent proliferation of the Glass^+^ daughter. In *Notch* mutants, both daughter cells become Glass^+^ CC precursors followed by additional rapid symmetric divisions. Model 3 depicts the possibility that Notch signaling restricts the development of Glass^neg^ CC progenitors. In *Notch* mutants, increased numbers of Glass^neg^ CC progenitors (red) produce multiple Glass^+^ cells that are CC precursors.

The earliest known CC cell lineage marker *glass* is detected at embryonic stage 11 [9]; thus, we postulated that the requirement for *Delta* prior to this stage indicated that Notch signaling specifies the number of Glass^+^ CC precursors. Glass protein is first detected in AKH^neg^ CC cell precursors, a pair of single cells emerging near the dorsal head midline at early stage 11 (red arrowheads in Figure 3B) [11]. Between stages 11 and 13, the number of Glass^+^ CC precursors increases to 14–16 cells (comprised of two clusters of 7–8 cells; Figure 5A) that migrate posteriorly to become AKH^+^ CC cells [11]. To investigate the basis of CC cell expansion in *Delta*, *Notch*, and *E(spl)* mutants, we first examined the emergence of Glass^+^ CC precursors near the head midline. At stage 10, in both wild type and *Notch* mutant embryos, no Glass^+^ CC precursors were detectable (Figure 3A and 3E). Thus, the CC cell lineage did not develop precociously in *Notch* mutants. In early stage 11 (see Materials and Methods), the first pair of midline Glass^+^ CC precursors emerged in wild type embryos (red arrowheads in Figure 3B). In contrast, up to 6 Glass^+^ CC precursors were detectable in *Notch* mutants at early stage 11 (white arrowhead and insert in Figure 3F). We observed variant increases in left and right groups of Glass^+^ CC precursors at this stage (2 cells indicated by red arrowhead in Figure 3F). In mid stage 11, a pair of Glass^+^ CC precursors remained as single cells in wild type embryo (red arrowheads in Figure 3C) while clusters of 4–6 Glass^+^ CC precursors were detectable in *Notch* mutants (white and red arrowheads in Figure 3G). By late stage 11 in wild type embryo, CC precursors commenced division to increase the number of Glass^+^ cells from 1 to 2 (white arrowhead and insert in Figure 3D). Likewise, the number of Glass^+^ CC precursors increased from 6–7 to an average of 14 cells in late stage 11 *Notch* mutant embryos (18 cells indicated by white arrowhead and insert in Figure 3H). These findings suggest that the increase of AKH-expressing CC cells found in *Delta*, *Notch* and *E(spl)* mutants reflects emergence of extra Glass^+^ CC precursors at early stage 11.

Our analysis of static images did not preclude that a single Glass^neg^ progenitor or Glass^+^ CC precursor might continuously proliferate in early stage 11 to produce an expanded number of Glass^+^ CC precursors (Figure 3J, models 1 and 2). To evaluate this possibility, we continuously imaged live embryos expressing a *glass*5.2-RedHStinger reporter (*glass*5.2-RHS) with fluorescence microscopy (Video S1). Nuclear localized fluorescent protein produced from this reporter permitted detection and counting of emerging *glass*-expressing CC precursors in early stage 11 wild type embryos (Figure 3I ‘wild type’, white arrowhead at t = 0 minutes). The signal intensity of the *glass*5.2-RHS reporter continuously increased until late stage 11 when the CC precursor divided to produce two adjacent progeny cells with equivalent reporter emission intensity (Figure 3I, t = 60 and 80 minutes). In *Notch* mutants, we observed a different sequence of cell appearance and reporter labeling (Video S2). Two *glass*5.2-RHS^+^ cells initially emerged (Figure 3I ‘*Notch*’, white arrowheads at t = 0 minutes). 20 minutes later, three additional *glass*5.2-RHS^+^ cells appeared (Figure 3I, black arrowheads at t = 20 minutes in panels labeled ‘*Notch*’). The three *glass*5.2-RHS^+^ cells appearing at this later time are not adjacent to the first two *glass*5.2-RHS^+^ cells. The emission intensity of these ‘new’ cells is fainter than that of the initial two cells. Thus, it is unlikely these new cells which appeared within 10 minutes represent progeny of the first two *glass*5.2-RHS^+^ cells. At 50 minutes, two additional *glass*5.2-RHS^+^ cells appeared (Figure 3I ‘*Notch*’, red arrowheads at t = 50 minutes), resulting in seven CC precursors. As in wild type embryos, CC precursor division begins at 60 minutes, and by 80 minutes the number of *glass*5.2-RHS^+^ cells in the *Notch* mutant was doubled. The number and density of *glass*5.2-RHS^+^ cells in the *Notch* mutant precluded further imaging and analysis. Thus, we did not detect accelerated proliferation by the first CC precursors appearing in *Notch* mutants. Rather, these data suggest that emergence of excess Glass^+^ CC precursors from Glass^neg^ progenitors is the basis for CC cell expansion following disruption of Notch signaling (Figure 3J, model 3).

### Corpora cardiaca precursors originate from head mesoderm

**Figure 4:**
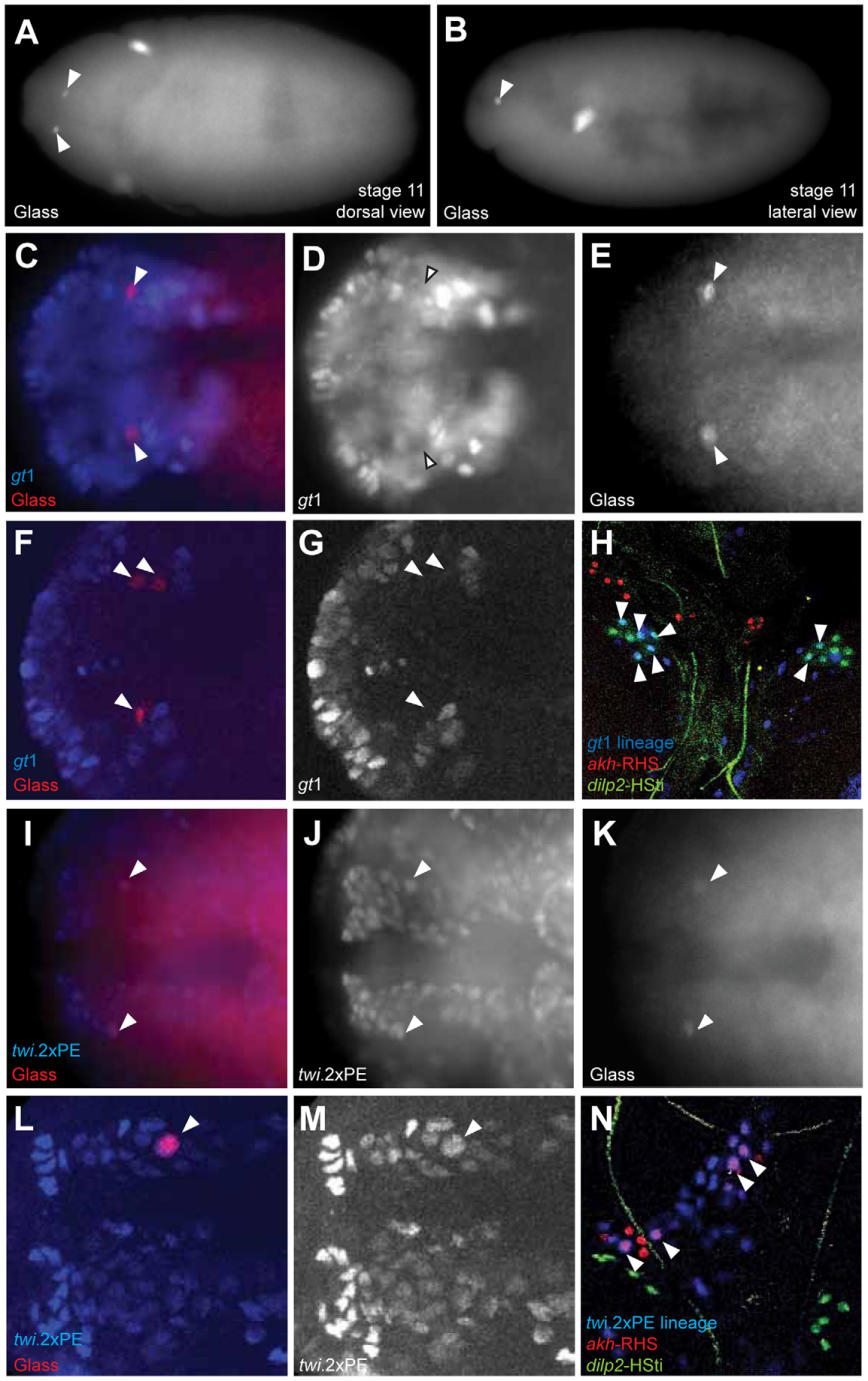
Copora cardiaca precursors originate from head mesoderm. (A–B) Locations of Glass^+^ CC precursor in stage 11 embryos in the dorsal view (A) and the lateral view (B). (C–E) *gt*1-GAL4; UAS-lacZ.NZ embryo expresses β-gal in anterior head neuroectoderm. *gt*1-GAL4 is expressed in the anterior head and several cells posterior to Glass^+^ CC precursors. *gt*1-GAL4 does not express in CC precursors as shown by the arrowheads. (F, G) Single confocal plane image of *gt*1-GAL4; UAS-lacZ.NZ embryonic head region stained for β-gal. Glass^+^ CC precursors (F, arrowheads) are not co-localized with *gt*1-GAL4 expression. (H) Lineage tracing of *gt*1-GAL4^+^ cells in third instar larval IPC and CC cells. IPC and CC cells are marked by *dilp2*-HSti (green) and *akh-*RHS (red) respectively. β-gal expression (blue) from *gt*1-GAL4; UAS-FLP; Act5C(FRT.polyA)lacZ.nls1 is co-localized with a subset of IPCs (green), resulting in IPCs with cyan (arrowheads). CC cells (red) are not labeled by *gt*1-GAL4 lineage tracing, and therefore do not show any cells in magenta. (I–K) Glass^+^ CC precursors in stage 11 embryo (arrowheads) are a part of the dorsal mesoderm marked by *twi*.2×PE-GAL4; UAS-lacZ.NZ. (I) Merged image shows that Glass^+^ cells are located in outer part of the head mesoderm. (J) β-gal expression marks the dorsal head mesoderm. (L, M) Single confocal plane image of *twi*.2×PE-GAL4 (M, β-gal) expression in a stage 11 embryo showing Glass^+^ CC precursor (L, arrowhead) originates from head mesoderm. (N) Lineage tracing of *twi*.2×PE-GAL4 expressing cells in third instar larval CC cells. Lineage was traced by β-gal expression (blue) in twi.2×PE-GAL4; UAS-FLP; Act5C(FRT.polyA)lacZ.nls1 larvae. *twi*.2×PE-GAL4 lineage-traced in several CC cells (magenta with arrowhead), but not in IPCs (green). All embryo images except (B) are stage 11 dorsal views with anterior to the left.

**Figure 5:**
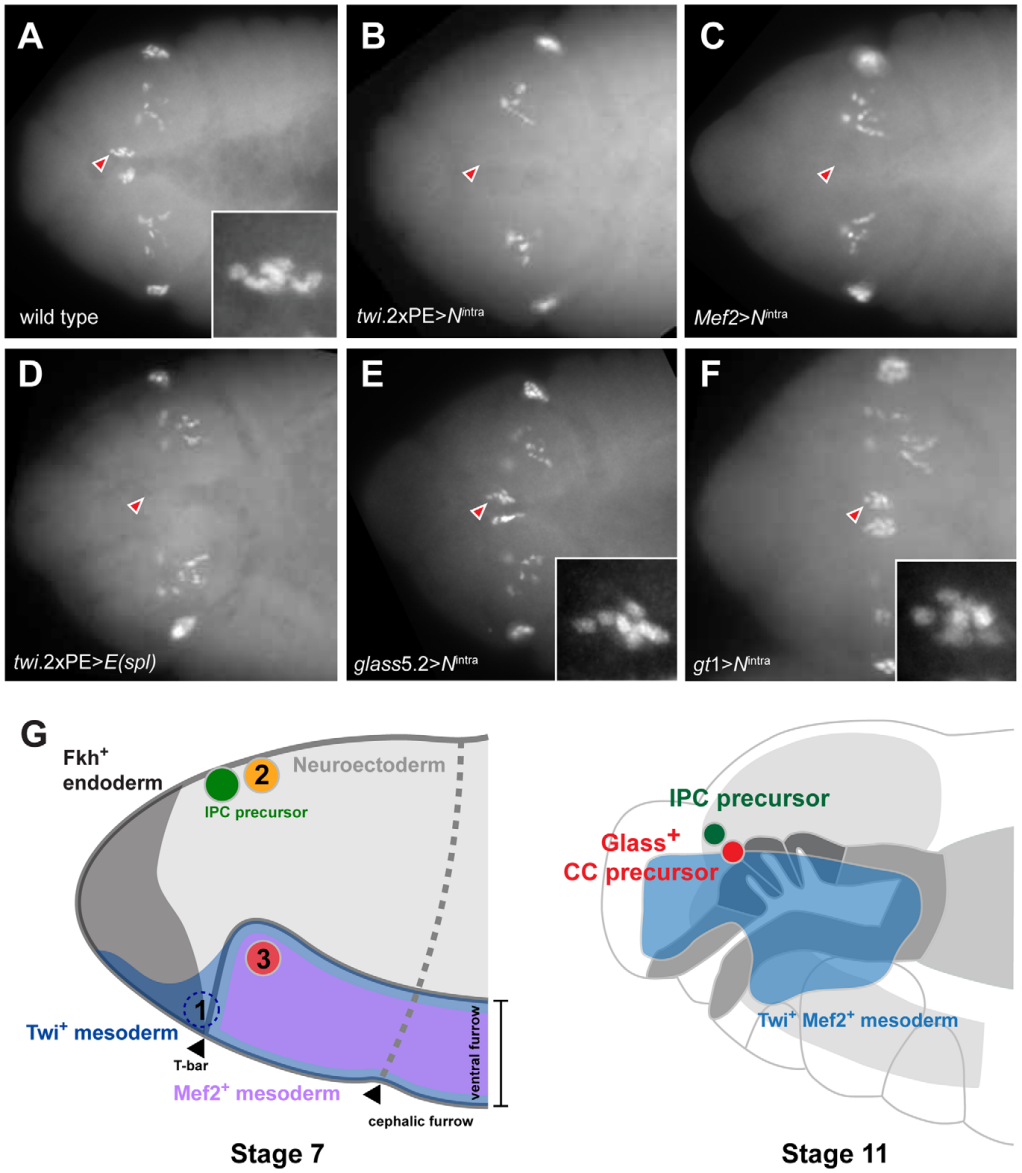
Ectopic activation of Notch signaling prior to Glass expression in head mesoderm disrupts CC precursor development. (A) Wild type embryo shows two clusters of Glass^+^ CC precursors, each one with 6 precursors (arrowhead and insert) in stage 12 embryo. Other Glass^+^ cells are larval eye precursors and brain primordia. (B) Activation of Notch signaling in mesoderm by *twi*.2×PE-GAL4; UAS-*N* ^intra^ removes the CC precursors (arrowhead) in stage 12 embryo. (C) Activation of Notch signaling in muscle lineage by *Mef2-*GAL4; UAS-*N* ^intra^ removes the CC precursors (arrowhead) in stage 12 embryo. (D) Ectopic expression of bHLH repressor *E(spl)* in mesoderm by *twi*.2×PE-GAL4; UAS-*E(spl)* also removes Glass^+^ CC precursors (arrowhead) in stage 12 embryo. (E) Activation of Notch signaling after Glass^+^ expression initiates in CC cell lineage does not perturb their development. *glass*5.2-GAL4; UAS-*N* ^intra^ embryo in stage 12 maintains normal number of Glass^+^ CC precursors (arrowhead and insert show 6 cells). (F) Activation of Notch signaling in neuroectoderm by *gt*1-GAL4; UAS-*N* ^intra^ does not disrupt the development of CC precursors (arrowhead and insert show 6 cells) in stage 12 embryo. All embryo images are dorsal views with anterior to the left. (G) Relative locations of CC and IPC precursors at stage 7 and 11 embryos. Embryos are drawn in the lateral view with anterior to the left. (1) *twi* ^+^ *gt* ^+^ cells, located in front of ventral furrow were proposed as an origin of CC cells by De Velasco et al [9], [10]. (2) Neighboring cells from the anterior neuroectoderm were proposed as origins for IPC and CC cells by Wang et al [11]. (3) The origin of CC cells identified by lineage tracing from Twist and Mef2 expressing head mesoderm in this study. By stage 11, Twi^+^ Mef2^+^ mesoderm has generated Glass^+^ CC precursors that are located near IPC precursors (green) and the endodermal foregut invagination (outlined by dark grey), where cells comprising the stomatogastric nervous system are born.

A recent study suggested CC cells develop from neuroectoderm [11] (site marked ‘2’ in Figure 5G), based on immunohistochemical detection of Glass in a subset of ectodermal cells labeled by a *giant*1-lacZ reporter (*gt*1-lacZ) [13]. With the goals of confirming this developmental origin, and controlling Notch signaling in progenitors of the CC cell lineage, we generated a *gt*1-GAL4 transgenic line (with an enhancer identical to the reported *gt*1-lacZ construct; see Materials and Methods). Anterior head expression of β-galactosidase (β-gal) in our *gt*1-GAL4; UAS-lacZ.NLS embryos (Figure S2D) was identical to the expression of *gt*1-lacZ expression reported previously (Figure S2A) [11]. However, the cytoplasmic β-gal signal from *gt1*-lacZ appeared diffuse, and was difficult to discern at single cell resolution (Figure S2A). Nuclear β-gal expression in *gt*1-GAL4; UAS-lacZ.NLS marked several cells near the Glass^+^ CC precursors (Figure 4A), but to our surprise we did not detect nuclear β-gal in Glass^+^ CC cell precursors in stage 11 embryos (Figure 4D). *gt*1-GAL4 cell lineage marking using FLP-recombinase (see Materials and Methods) traced *gt*1-GAL4 expression to third instar larval IPCs marked by the *dilp2*-HSti reporter (arrowheads in Figure 4H). This result is consistent with the reported origin of IPCs from *gt*1-lacZ expressing cells [11], and validates use of *gt*1-GAL4 for lineage tracing. However, *gt*1-GAL4 cell lineage marking did not trace to larval CC cells expressing the *akh*-RHS reporter (red in Figure 4H), showing that CC cells do not originate from *gt*1-expressing head neuroectoderm.

Based on expression and mutant phenotype analysis of genes that expressed in embryonic head, De Velasco et al [9], [10] suggested that CC cells originate from cells adjacent to the anterior ventral furrow (site marked ‘1’ in Figure 5G). To test if CC cells derive from *twist-*expressing mesoderm cells at this anterior junction between embryonic endoderm and mesoderm, we used the *twi*.2×PE-GAL4 line to label progeny of 12–14 ventral most mesodermal cells, as previously described [14]. Nuclei of the mesodermal cells and their progeny were labeled with β-gal through stage 11 in *twi*.2×PE-GAL4; UAS-lacZ.NLS embryos (Figure 4J). A subset of these β-gal^+^ mesodermal progeny co-expressed Glass (Figure 4I). Thus, ventral *twist-*expressing mesodermal cells invaginate and migrate toward the dorsal midline where Glass^+^ CC precursors are specified (blue domain in Figure 5G). In third instar larvae, lineage tracing of *twi*.2×PE-GAL4^+^ cells using FLP-recombinase revealed nuclear localization of β-gal in the majority of *akh-*RHS^+^ CC cells (arrowheads in Figure 4N). By contrast, IPCs were always β-gal^neg^ (green nuclei in Figure 4N). We also used *Mef2-*GAL4 line to trace embryonic and larval lineages derived from all muscle lineages beginning at stage 7 embryos [15] (purple domain in Figure 5G). Similar to our findings with *twi*.2×PE-GAL4, we observed labeling of Glass^+^ CC precursors with *Mef2*-GAL4; UAS-lacZ.NLS at stage 11 (Figure S3A–S3C), and labeling of mature larval AKH^+^ CC cells with *Mef2* lineage tracing (arrowheads in Figure S3D). These results demonstrate that Glass^+^ CC precursors originate from head mesoderm, and that IPC and CC cells derive from distinct germ layers in *Drosophila*.

To test this conclusion further, we asked if impaired CC development resulted from Notch signaling disruption in head mesoderm that expressed *twist* or *Mef2*. Based on our disruption of Notch signaling using loss-of-function or conditional mutations, we postulated that head mesodermal expression of the *N* ^intra^ allele, which encodes a constitutively activate form of *Notch* [16], or *E(spl)* prior to stage 11 should reduce or eliminate development of embryonic stage 12 Glass^+^ CC precursors. By contrast, Notch signaling activation after formation of Glass^+^ CC precursors should not impair subsequent CC development. In *twi*.2×PE-GAL4; UAS-*N* ^intra^ embryos and in *Mef2*-GAL4; UAS- *N* ^intra^ embryos, we failed to detect Glass^+^ CC precursors (Figure 5B and 5C), confirming that CC cells originate from mesoderm that expresses *twist* and *Mef2*. Likewise, we observed elimination of Glass^+^ CC precursors in stage 12 *twi*.2×PE-GAL4, UAS-*E(spl)* embryos (Figure 5D). In contrast to these results, the number of Glass^+^ CC precursors at stage 12 was not detectably altered in *glass*5.2-GAL4; UAS-*N* ^intra^ embryos compared to control embryos (Figure 5E). Thus, consistent with our studies of the conditional *Delta* ^RF^ mutants, these results indicate that Notch signaling may be dispensable after *glass*-expressing CC precursors are established at the early stage 11. To test if activation of Notch signaling in neuroectoderm affects CC development in the adjacent mesoderm, *N* ^intra^ was expressed in head neuroectoderm by *gt*1-GAL4. The number of Glass^+^ CC precursors at stage 12 was not altered in *gt*1-GAL4; UAS-*N* ^intra^ embryos (Figure 5F), suggesting that Glass^+^ CC precursors develop independently of Notch signaling in neuroectoderm. Taken together, these results argue that CC cells originate from head mesoderm.

### *daughterless* and *tinman* are required for CC cell development

**Figure 6:**
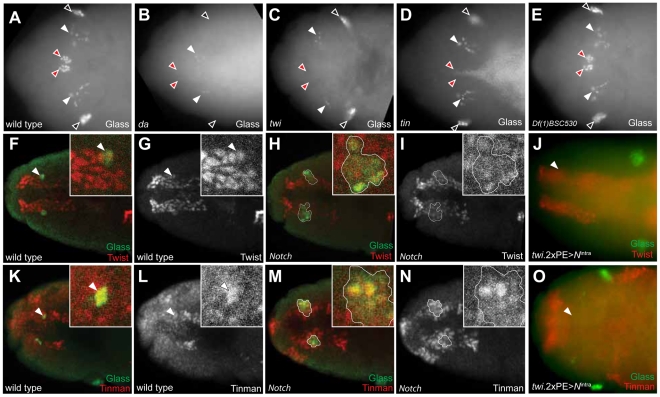
Tinman expression in head mesoderm is regulated by Notch signaling. (A) Glass expression in stage 12 wild type embryonic head region. CC precursors are located in the dorsal midline (red arrowheads). Black arrowheads indicate Glass^+^ larval eye precursors while white arrowheads indicate Glass^+^ brain primordia. (B) In *da* mutant at stage 12, both CC (red arrowheads) and eye precursors (black arrowheads) are missing, but Glass expression in the brain primordia (white arrowheads) is maintained. (C) In *twi* mutant at stage 12, CC precursors are missing in the dorsal midline (red arrowheads) while brain (white arrowheads) and eye precursors (black arrowheads) are intact. (D) In *tin* mutant at stage 12, CC precursors are missing in the dorsal midline (red arrowheads). (E) In stage12 *Df(1)BSC530* mutant in which proneural genes are removed, CC precursors (red arrowheads) are intact. (F, G) Dorsal view of stage 11 wild type embryonic head shows Glass^+^ CC precursors (arrowhead and green in insert of F) co-localized with Twist^+^ cells (red). (H, I) Dorsal view of stage 11 *Notch* mutant shows multiple Glass^+^ CC precursors (outlined and green in insert) co-localized with Twist^+^ cells (red). (J) Twi expression in head mesoderm is maintained in stage 11 embryo when Notch signaling is activated in mesoderm by *twi.*2×PE-GAL4; UAS-*N* ^intra^. (K, L) Dorsal view of stage 11 wild type embryonic head shows Glass^+^ CC precursors (arrowhead and green in K) co-localized with Tinman^+^ cells (red). (M, N) Dorsal view of stage 11 *Notch* mutant shows multiple Glass^+^ CC precursors (outlined and green in insert) co-localized with Tinman^+^ cells (red). (O) Both Glass and Tinman expression in head mesoderm is abolished at stage 11 embryo when Notch signaling is activated in mesoderm by *twi.*2×PE-GAL4; UAS-*N* ^intra^. All embryo images are dorsal views with anterior to the left.

During trunk mesoderm development, bHLH transcription factors, encoded by *daughterless* (*da*) and *twist* (*twi*), are necessary for the allocation of mesodermal cells to specific fates [17]–[19]. Prior study showed Twist is required for CC development [9], but it was not known if *daughterless* or specific Twist targets were required for CC development. Thus, we assessed requirements for Daughterless and Twist targets in CC cell development from head mesoderm. In late stage 12 wild type embryos, two groups of 6–7 Glass^+^ CC precursors are detectable near the dorsal midline (red arrowhead in Figure 6A). In stage 12 mutants lacking *daughterless* or *twist*, these Glass^+^ precursors are absent (red arrowheads in Figure 6B, 6C). Twist regulates expression of several transcription factors required for trunk mesoderm differentiation, including *Zn finger homeodomain 1* (*zfh1*), *Myocyte enhancer factor 2* (*Mef2*), *held out wings* (*how*), and *tinman* (*tin*) [20]–[25]. To test if these transcription factors are also required for CC development, we assessed CC precursor development in mutant embryos. Normal numbers of CC precursors were detected in embryos harboring mutations in *zfh1*, *Mef2*, and *how* (data not shown). By contrast, Glass^+^ CC precursors were not detected in stage 12 embryos with mutations in *tinman* (red arrowheads, Figure 6D). Since proneural genes are required for stomatogastric nerve cell precursor formation [8] and specification of muscle progenitors [26], we also tested mutant embryos deficient for the genes *achaete*, *scute*, *lethal of scute*, and *asense*, which encode proneural bHLH factors. However, the number of CC precursors was not altered in mutant embryos (Figure 6E), suggesting these proneural genes are not required for CC development. Thus, our mutant analysis revealed a specific requirement for *twist*, *daughterless*, and *tinman* transcription factors during CC precursor specification from head mesoderm.

### Tinman expression in head mesoderm is regulated by Notch signaling

We first postulated that regulation of *twist* expression by Notch signaling in head mesoderm, like in trunk mesoderm [27], might underlie CC cell expansion in *Notch* mutant flies. In wild type embryos, Twist expression by immunostaining was restricted to head mesoderm, and Glass^+^ CC precursors co-expressed Twist (inserts and arrowheads in Figure 6F, 6G). In stage 11 *Notch* mutants, we observed that two clusters of multiple Glass^+^ CC precursors co-localized with these Twist^+^ cells (one cluster enlarged in Figure 6H, 6I), indicating that the multiple Glass^+^ CC precursors in *Notch* mutants originate also from Twist^+^ head mesoderm. To test whether head mesodermal Twist expression may be regulated by Notch signaling, we asked if the ectopic activation of Notch signaling in head mesoderm in stage 11 *twi*.2×PE-GAL4; UAS-*N* ^intra^ embryos results in loss or reduction of Twist expression. A normal pattern of Twist expression in head mesoderm was observed in these embryos but Glass^+^ CC precursors were absent (arrowhead in Figure 6J), providing additional evidence that the level of Twist expression in head mesoderm may not be regulated by Notch signaling.

Tinman expression is restricted to the anterior dorsal region of head mesoderm in stage 11 wild type embryos (Figure 6L for dorsal view and Figure S4C for lateral view), and Tinman^+^ head mesodermal cells at stage 11 include Glass^+^ CC precursors (arrowheads and enlarged in Figure 6K, 6L, and Figure S4A). In stage 12 embryos, Tinman expression in Glass^+^ CC precursors was extinguished, while adjacent Glass^neg^ cells - which include the procephalic vascular rudiment [10] - maintained Tinman expression (Figure S4E–S4H). In *Notch* mutants, the number of Glass^+^ Tinman^+^ CC precursors in head mesoderm increased (outlined in Figure 6M and 6N). In addition, Glass^neg^ Tinman^+^ cells adjacent to Glass^+^ CC precursors also appear expanded (brackets in Figure S4J and S4L), suggesting that Tinman expression in head mesoderm may be regulated by Notch signaling, To test this possibility, we asked if ectopic Notch signaling activation in head mesoderm resulted in loss of Tinman expression. Expression of both Glass and Tinman was abolished in the head mesoderm of *twi*.2×PE-GAL4; UAS-*N* ^intra^ embryos (arrowhead in Figure 6O). Together, these results show that Tinman expression is regulated by Notch signaling in head mesoderm, and suggest the possibility that *tinman* mis-expression in this context underlies CC lineage expansion in Notch signaling disruption.

### Co-expression of Tinman and Daughterless in mesoderm is sufficient for CC cell lineage specification

**Figure 7:**
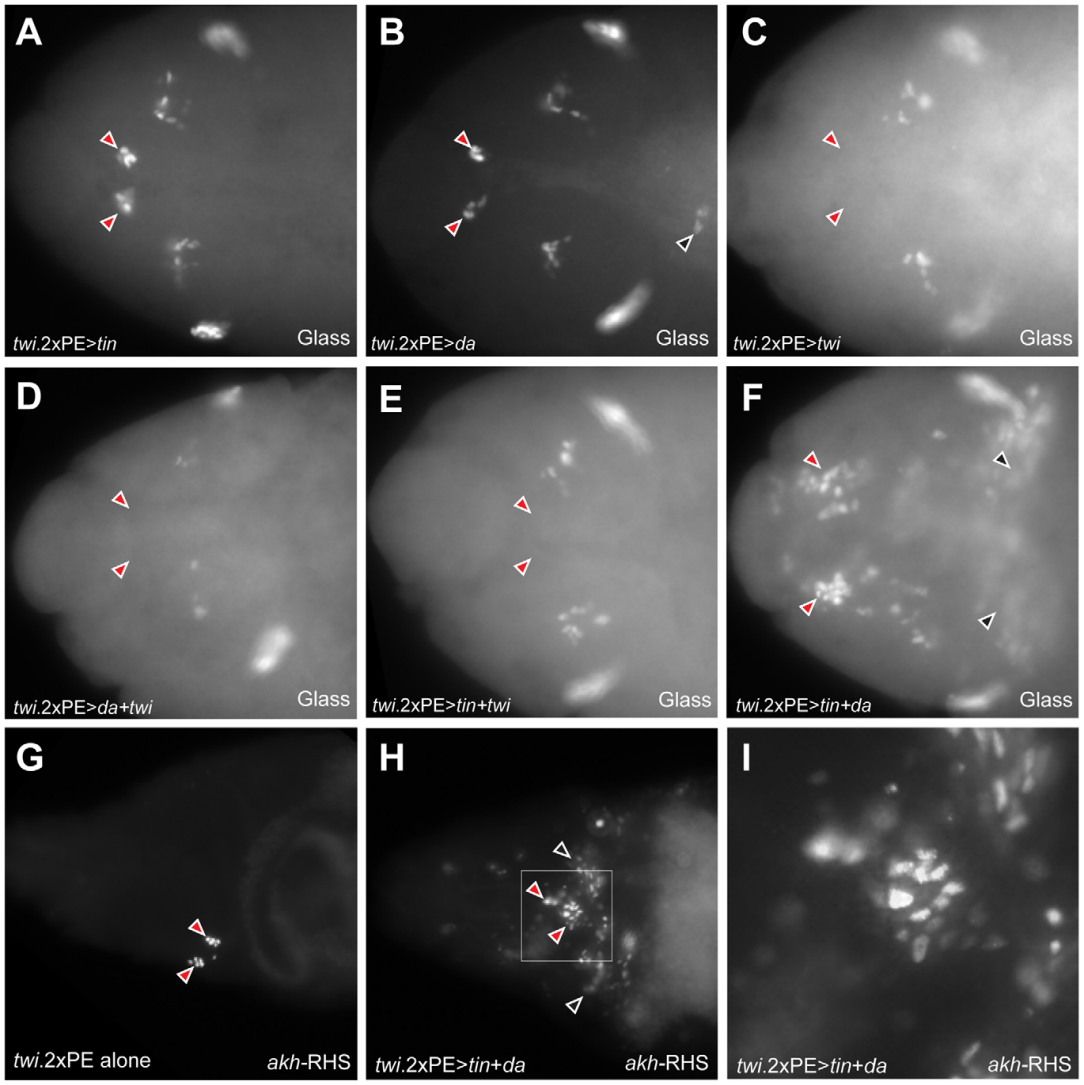
Co-expression of Daughterless and Tinman in mesoderm is sufficient for ectopic CC cell lineage specification in mesoderm. (A) Glass^+^ CC precursors are not expanded (red arrowheads) in stage 12 *twi.*2×PE-GAL4 UAS-*tinman* embryo. (B) CC precursors (red arrowheads) are developed normally in stage 12 *twi.*2×PE-GAL4 UAS-*daughterless* embryos. Ectopic Glass expressing cells in trunk region is marked by black arrowhead. (C–E) CC precursors are absent (read arrowheads) when *twist* is over-expressed alone (C) or *twist* is co-expressed with *daughterless* (D) or *tinman* (E) in head mesoderm. (F) Glass^+^ CC precursors in head mesoderm are expanded in stage 12 *twi.*2×PE-GAL4 UAS-*tinman* UAS-*daughterless* embryo (red arrowheads). Ectopic Glass expressing cells are also detected in trunk region (black arrowheads). (G) CC cells are marked by *akh-*RHS expression in stage 17 control embryo. (H) Expansion of CC cells (red arrowheads) and ectopic CC cells in trunk region (black arrowheads) are detected in stage 17 *twi.*2×PE-GAL4 UAS-*tinman* UAS-*daughterless* embryo. (I) Magnified view of a box marked in (H) to show the expansion of *akh-*RHS expressing CC cells. All embryo images are dorsal views with anterior to the left.

Since expanded Tinman expression in *Notch* mutant head mesoderm accompanied CC lineage expansion, we investigated if ectopic expression of *tinman* might be sufficient to expand CC cells. However, in *twi.*2×PE-GAL4 UAS-*tinman* embryos, the population of Glass^+^ CC precursors was not expanded (Figure 7A), suggesting that additional factors may be required to specify the CC cell lineage in head mesoderm. During trunk mesoderm differentiation, Twist activity is inhibited by its dimerization partner Daughterless to allocate mesodermal cells to various tissue fates [19]. Therefore, we next investigated effects of mis-expressing *daughterless* or *twist* in mesoderm. In *twi.*2×PE-GAL4 UAS-*daughterless* embryos, Glass^+^ CC precursors do not increase in head mesoderm (Figure 7B), although we reproducibly observed appearance of ectopic Glass^+^ cells in the trunk of these embryos (black arrowhead in Figure 7B). In contrast, Glass^+^ CC precursors were absent in *twi.*2×PE-GAL4 UAS-*twist* embryos (Figure 7C), suggesting that CC precursor specification is inhibited by excessive Twist activity. Ectopic CC cell development from mis-expression of *daughterless* or *tinman* in head mesoderm was also eliminated by co-expression of *twist* (Figure 7D, 7E), supporting the view that excess Twist activity can suppress CC development.

Daughterless protein contains a repression domain, and can heterodimerize with Twist to regulate Twist activity [28]. Thus, we postulated that CC cell lineage specification may be regulated by *tinman* in mesodermal cells with increased Daughterless activity. To test this possibility, we co-expressed *tinman* with *daughterless* in mesoderm. In *twi.*2×PE-GAL4 UAS-*tinman* UAS-*daughterless* embryos, the number of Glass^+^ CC precursors in head mesoderm was markedly expanded (red arrowheads in Figure 7F). In addition to extra Glass^+^ cells in head mesoderm, we also detected ectopic Glass^+^ cells in the trunk (black arrowheads in Figure 7F). To test if these Glass^+^ cells developed further toward a fate resembling CC cells, we assessed *akh*-RHS marker expression at stage 17. Compared to normal *akh-*RHS^+^ cell numbers in *twi.*2×PE-GAL4 control embryos at stage 17 (Figure 7G), we detected increased numbers of *akh-*RHS^+^ cells in *twi.*2×PE-GAL4 UAS-*daughterless* UAS-*tinman* embryos at this stage (red arrowheads in Figure 7H). Unexpectedly, we also detected ectopic *akh-*RHS^+^ cells in embryonic trunk of these embryos at stage 17 (black arrowheads in Figure 7H). Taken together, these results show that co-expression of Daughterless and Tinman is sufficient to activate CC cell developmental programs and to promote CC cell lineage expansion both in head mesoderm and ectopic sites. Collectively, the results strongly suggest that the CC cell lineage is specified by a combinatorial transcription code in embryonic mesoderm.

## Discussion

### Identification of novel genes required for CC cell development

Although DrosDel deficiency lines used in this study cover only ∼50% of *Drosophila* genome, we successfully identified several genes previously not implicated in CC cell development. Mutations in *crooked neck (crn)*, *spitz (spi)*, *dimmed (dimm)*, *phyllopod (phyl)*, *double parked (dup)*, *three rows (thr)*, *Polycomblike (Pcl)*, *ETS-domain lacking (edl)*, and *heartless (htl)* result in the complete loss of *Akh* expression. Expression of *dimm* in CC cells has been previously reported [29], and *dimm* is required for the differentiation of central and peripheral neuroendocrine cells. Thus, *dimm* may be required for CC cell maturation. *spi*, *edl*, and *phyl* are components of the Epidermal Growth Factor signaling pathway and *htl* encodes a *Drosophila* Fibroblast Growth Factor Receptor. Thus, these results suggest that MAPK signaling pathways regulate CC cell development. *thr*, *dup*, and *crn* are required for the cell cycle control, suggesting that the regulation of cell cycle control is also important for proper CC cell development.

### Disruption of Notch signaling leads to the expansion of neuroendocrine precursor cells

Prior studies suggest that development of stomatogastric endocrine cells from endoderm, and IPCs from neuroectoderm is regulated by Notch and MAPK signaling [30]–[32]. Here, we found that Notch signaling disruption from mutations in *Notch*, *Delta* or *E(spl)* led to expansion of CC cells, reminiscent of the expansion of endocrine islet α-cells during mammalian pancreas development of *Dll1* or *Hes1* mutant mice [33], [34]. Notch signaling is required to maintain undifferentiated mammalian pituitary progenitors (reviewed in [35]), and mutations disrupting Notch signaling also result in the expansion of specific pituitary cell types [36]. Thus, signaling pathways controlling CC cell development may reflect ancient conserved genetic programs for endocrine cell specification. Using time-lapse *in vivo* imaging, we detected the emergence of multiple Glass^+^ CC precursors in stage 11 *Notch* mutants. The most rapid mitotic divisions in *Drosophila* occur prior to embryonic cellularization, and require approximately 10 minutes [37]. Thus, we calculate that the emergence of 7 Glass^+^ CC precursors within 20 minutes in *Notch* mutant embryos is unlikely to result from accelerated division of a single Glass^+^ CC precursor or loss of asymmetric cell division from Glass^neg^ CC progenitor. Rather, our data suggest that Notch signaling restricts head mesodermal fate specification possibly by a lateral inhibition mechanism (model 3 in Figure 3J). After the initial Glass^+^ CC precursors are formed, maturation process from a single Glass^+^ CC precursor to a cluster of 7–8 AKH^+^ CC cells appears to be Notch signaling independent. Conditional mutant studies using a temperature sensitive allele of *Delta*, or using Notch signaling activation in Glass^+^ CC precursor cells further support this possibility.

### Mesodermal origin of neuroendocrine cells

Prior studies suggested that corpora cardiaca neuroendocrine cells in *Drosophila* may derive from the most anterior region of head mesoderm expressing *twist* and *gt* [9], [10]. Recently, an alternate neuroectodermal origin for CC cells was proposed [11]. CC cells manifest neuron-like features, lending plausibility to the suggestion that CC cells derived from neuroectoderm expressing *gt*1-lacZ. However our study identified that the corpora cardiaca originates from head mesoderm expressing *twist*, *Mef2* and *tinman*. The absence of CC precursors in *twist* and *tinman* mutants also strongly support this view. Lineage tracing studies by cell marking with *gt*1-GAL4 here confirmed a neuroectodermal origin for insulin-producing neurons in the protocerebrum; however, we did not detect tracing of CC precursors or mature CC cells from *gt*1-expressing cells. Thus, CC cells and IPCs have distinct embryonic origins and our data provide conclusive evidence from lineage tracing that neuroendocrine CC cells derive from mesoderm. The origins of IPCs and CC cells from different germ layers is consistent with the observation that mutations preventing CC cell development do not detectably impair IPC formation [38]. Thus, cell interactions between IPCs and CC cells may not be essential for development of these two cell types. A prior study speculated that corpus allatum cells in the larval ring gland, which produce juvenile hormone, derive from gnathal mesoderm [9], but this origin has not been demonstrated with methods like lineage tracing. Thus, to our knowledge, CC cells may represent the sole example, thus far, of neuroendocrine cell development from mesoderm in *Drosophila*.

Vascular access and dispersion of hormones is a defining feature of endocrine organs. In mammals, signaling between vascular and endocrine progenitors is an important mechanism for regulating development of organs like the pancreas [39]. Tinman^+^ cells in *Drosophila* head mesoderm also form the procephalic vascular rudiment [10], whose progeny establish the contractile dorsal vessel (*Drosophila* heart), and prior studies have demonstrated that axon-like projections from larval CC cells terminate on the dorsal vessel [3]. In addition, similar to the posterior migration of head-mesodermal rudimentary vascular cells, Glass^+^ AKH^neg^ CC progenitors migrate posteriorly during their maturation into AKH^+^ cells. De Velasco and colleagues have previously speculated that developing CC precursors might interact with other head mesoderm cells [9] during CC development. Our demonstration that CC cells originate from Tinman^+^ Glass^neg^ head mesoderm further supports this possibility. The proximity of embryonic CC cell progenitors to dorsal vessel progenitors may enhance cell-cell interactions that govern hallmark CC cell properties, including AKH expression and physical connections to their vascular targets. Together, these observations suggest that key morphogenetic and developmental signaling relationships between endocrine and vascular precursors may be conserved from flies to mammals.

### Encoding neuroendocrine lineage specification by transcription factor combinations

Many human diseases result from excessive or inadequate endocrine cell mass or function. Thus, there is intense interest in identifying evolutionarily-conserved transcriptional codes for neuroendocrine cell development and expansion. Our study identified a unique cell signaling context in mesoderm where neuroendocrine precursor cells can be specified by the two transcription factors Tinman and Daughterless. Allocation of trunk mesodermal fates is regulated by Twist and Daughterless activity [19], [28], and here we showed that CC cell specification in head mesoderm is also regulated by a combination of transcription factors. *tinman* expression in a small subset of head mesoderm is regulated by Notch signaling, reminiscent of *tinman* regulation in trunk cardiogenic mesoderm by Notch signaling [40]. However, only two cells within Tinman^+^ domain in head mesoderm develop into Glass^+^ CC progenitors. These observations suggest that other factors, in addition to Tinman, are required to specify the CC cell lineage. Consistent with this possibility, we show that Tinman mis-expression is not sufficient to expand CC development. By contrast, co-expression of Tinman and Daughterless led to increased development of head mesoderm into CC cells; thus, Tinman and Daughterless collaborate to specify the CC lineage. The combination of Tinman and Daughterless also induced ectopic AKH^+^ cells in the embryonic trunk, suggesting that trunk mesodermal cells may also be competent to develop into CC cells. We speculate that over-expression of Daughterless in mesoderm suppresses Twist activity, and the mesodermal cells in this context are competent to become CC lineage upon Tinman expression, but further studies are required to test this possibility. Our study identified a transcription factor combination whose reconstitution is sufficient for differentiation by a subset of mesodermal cells toward a neuroendocrine fate. However, most embryonic mesodermal cells failed to express Glass or *Akh* upon mis-expression of Tinman and Daughterless, suggesting additional factors are likely required to re-specify mesoderm into CC cells. Moreover, additional studies are needed to determine how Daughterless, which is ubiquitously expressed, might regulate Twist activity in differentiating mesoderm to give rise to distinct cell fates.

In summary, work here reveals embryonic and molecular mechanisms regulating development of *Drosophila* CC cells. We demonstrated that Notch signaling restricts CC precursor cell fate in head mesoderm and regulates Tinman expression. We used cell lineage tracing and genetic analysis to demonstrate that CC cells originate from embryonic mesoderm. We also showed that a combination of the transcription factors Tinman and Daughterless is necessary and sufficient to specify CC cell lineage in mesoderm. Findings from this study should accelerate advances in our understanding of the conserved molecular mechanisms controlling differentiation and expansion of endocrine organs essential for metabolic regulation.

## Materials and Methods

### *Drosophila* strains

*y* ^1^ *w* ^1118^ strain was used as the wild type stock. DrosDel deficiency lines were obtained from Bloomington Stock Center. The following mutant alleles and transgenic lines were used in this study: *Dl* ^9P^, *Dl* ^RF^, *N* ^264-39^, *E(spl)* ^rv1^, *Ser* ^RX82^, *Su(H)* ^IB115^, *da* ^10^, *twi* ^1^, *zfh1* ^00865^, *Mef2* ^X1^, *how* ^stru-3R-3^, *Df(1)BSC530*, *twi.*2×PE-GAL4, GAL4-*Mef2.*R and UAS-lacZ.NZ (Bloomington Stock Center). *gt*1-lacZ was provided by Dr. Stephen Small (New York University) [13]. *tin* ^346^, *tin* ^EC40^ and UAS-*tin* were provided by Dr. Rolf Bodmer (Burnham Institute) [41]. UAS-*da* and UAs-*twi* were provided by Dr. Mary K. Baylies (Sloan-Kettering Institute) [19]. UAS*-N* ^intra^ was a gift from Dr. Margaret Fuller (Stanford University). *Kr*-GAL4 UAS-GFP or *twi*-GAL4 UAS-GFP harboring balancer chromosomes were used to identify hemi- or homozygous mutant embryos. For lineage tracing experiments, flies carrying UAS-FLP; *dilp2*-HSti, *akh-*RHS; *Act5C*(FRT.polyA)lacZ.nls1 were crossed to GAL4 lines.

### *In situ* hybridization and immunohistochemistry

Antisense riboprobe for *Akh* was derived from pBS2KSP-Akh cDNA clone. RNA *in situ* hybridization was carried out as described [42]. Immunostaining of embryos was performed as described [42] with the following modifications; all embryos were manually devitellinized to avoid methanol exposure, late stage 17 embryos with cuticle were sonicated for 6 seconds under the lowest output setting in Branson Sonifier 450, primary antibodies were detected with Alexa488, 547, or 647 (Invitrogen) secondary antibodies, and embryos were mounted in 100% glycerol. Embryonic developmental stages were morphologically determined according to Campos-Ortega and Hartenstein [43]. During our studies, we found that the development of Glass^+^ larval eye precursors in Bolwig's organ lineage was unaffected in *Notch* mutants, and we quantified Glass^+^ larval eye precursors to determine embryonic stage accurately within stage 11 embryos. In both wild type and mutant embryos, we detected 1–3 precursors at early stage 11 (black arrowheads in Figure 3B and 3F), 4–7 precursors at mid stage 11 (black arrowheads in Figure 3C and 3G) and 8–11 cells at late stage 11(black arrowheads in Figure 3D and 3H), respectively. The following primary antibodies were used: rabbit anti-AKH (1∶300) [3], rabbit anti-Twist (1∶500; Dr. Maria Leptin, Universität Köln) [17], rabbit anti-Tinman (1∶300; Dr. Manfred Frasch, Mount Sinai School of Medicine) [24], mouse 9B2.1c anti-Glass (1∶10; Developmental Studies Hybridoma Bank under the auspices of NICHD and maintained by The University of Iowa, Department of Biology) [44] and chicken anti-β-gal (1∶1000; Abcam). Immunostaining of CC cells in larval brains was performed as described [3]. Imaging of RNA *in situ* hybridizations was performed on a Zeiss Axio Imager DIC microscope. Immunofluorescence microscopy was performed on a Zeiss Axio Imager or a Zeiss LSM510 confocal microscope. Z-projections of confocal stacks were generated using ImageJ with sum slice option.

### Generation of reporter and GAL4 driver lines

The enhancer sequences used in this study were amplified from *y* ^1^ *w* ^1118^ genomic DNA. pAkhp1016 Red H-Stinger (*akh*-RHS) and pAkhp1016 Green H-Pelican (*akh-*GHP) were constructed by subcloning the 1016 bp sequence upstream of the *Akh* start codon [3] into the pRed H-Stinger and pGreen H-Pelican vectors [45], respectively. pDilp215-1-H-Stinger (*dilp2*-HSti) was generated by subcloning the 541 bp sequence upstream of the *dilp2* transcription start site [2] into the pH-Stinger vector. pGlass5.2 Red H-Stinger (*glass5.2*-RHS) was constructed by subcloning the 5197 bp sequence upstream of the *glass* start codon [46] into the pRed H-Stinger vector. pGt1-GAL4 (*gt*1-GAL4) was constructed by subcloning the 787 bp *gt*1 CRM fragment [13] into pPTGAL. pGlass5.2-GAL4 (*glass*5.2-GAL4) was constructed by subcloning this 5197 bp sequence into the pPTGAL vector. *P*-element mediated germline transformations were carried out as described [47]. For all transgenic strains, at least two independently-derived transgenic lines with transgenes mapping to the second or third chromosome were evaluated.

### Live embryo imaging

To capture fluorescent reporter signals in developing embryos, stage 7 or 8 embryos were mounted between two cover glasses spaced with 0.1% agarose blocks. Z-stack images (35×1 µm) were captured every 2 minutes for 3 hours in the Zeiss Axio Imager fluorescent microscope. Conversions of Z-stacks to projection images and time-lapse movies were performed in ImageJ software.

## Supporting Information

Figure S1 *Delta* regulates CC cell number before embryonic stage 11. (A) Temperature shift conditions applied to *Delta* ^RF^ mutants at different time points following a 1-hour egg lay. hAEL is ‘hours after egg lay’. 18°C is the permissive temperature, and 29°C is the restrictive temperature for *Delta* ^RF^ mutants. (B) *Delta* ^RF^ mutant grown at 18°C shows normal CC cell appearance, indicated by normal *akh-*GHP expression (arrowheads). (C–D) *Delta* ^RF^ mutant grown at 29°C for 3 hours (C) or 4 hours (D) followed by a shift to 18°C until stage 17 shows normal CC cell appearance (arrowheads). (E) *Delta* ^RF^ mutant shifted from 29 to 18°C at 5 hAEL showing normal CC cell development, accompanied by an anterior shift of CC cell position. (F) *Delta* ^RF^ mutant with a temperature shift at 6 hAEL shows a modest CC expansion. (G–H) *Delta* ^RF^ mutant with a temperature shift at 7 hAEL (G) or 8 hAEL (H) shows clear CC expansion. (I) *Delta* ^RF^ mutant grown continuously at 29°C exhibits *akh*-GHP^+^ CC expansion. All panels show dorsal views of embryos at late stage 17, with anterior to the left.
(TIF)

Figure S2 β-gal expression pattern comparison of *gt*1-lacZ and *gt*1-GAL4 UAS-lacZ.NZ in anterior head neuroectoderm. (A–C) Expression of β-gal (A) and Glass (B) in the anterior head of stage 11 *gt*1-lacZ embryo. (D–F) Expression of β-gal (D) and Glass (E) in the anterior head of stage 11 *gt*1-GAL4 UAS-lacZ.NZ embryo. Arrowheads mark Glass expressing CC precursors (B–C and E–F) and their locations (A and D).
(TIF)

Figure S3 CC cells originate from *Mef2*-GAL4 expressing mesoderm. (A–C) Glass^+^ CC precursors in stage 11 embryos are a part of *Mef2*-GAL4^+^ cells. The dorsal head mesoderm marked by *Mef2*-GAL4 expression (A, arrowhead), and Glass^+^ CC precursors (B, arrowhead) are co-localized (C, magenta cells with arrowheads) with ß-gal^+^ cells in *Mef2-*GAL4; UAS-LacZ.NZ embryos. (D) Lineage tracing of *Mef2*-GAL4^+^ cells in third instar larval CC cells. Lineage was traced by ß-gal expression (blue) in *Mef2*-GAL4; UAS-FLP; Act5C(FRT.polyA)lacZ.nls1 larvae. Several CC cells (red) are lineage-traced by *Mef2-*GAL4 expression (magenta cells with arrowheads). All embryo images are stage 11 dorsal views with anterior to the left.
(TIF)

Figure S4 Tinman expression in stage 11 and 12 embryonic head mesoderm. (A–D) Glass^+^ CC precursors (B) in stage 11 embryos are co-localized with Tinman^+^ (C) mesodem marked by *twi.*2×PE-GAL4 UAS-lacZ.NZ (D). The inserts show the enlarged area marked by a box in (A). (E–H) Glass^+^ CC precursors (F) lose Tinman expression (G), but maintain mesoderm marker (H) shown by *twi.*2×PE-GAL4 UAS-lacZ.NZ expression. (I, J) Dorsal view of stage 11 wild type embryonic head shows Glass^+^ CC precursors (arrowhead in I) co-localized with Tinman^+^ cells (red). The red bracket indicates the width of the Tinman^+^ cell cluster in wildtype head mesoderm. (K, L) Dorsal view of stage 11 *Notch* mutant shows multiple Glass^+^ CC precursors (outlined in insert in K) co-localized with Tinman^+^ cells (red). The bracket indicates the width of the Tinman^+^ cell cluster in *Notch* mutant head mesoderm. All embryo images are lateral views with anterior to the left.
(TIF)

Table S1 Identified DrosDel deficiency lines and genetic loci in which mutations resulted in altered number of CC cells.
(DOC)

Video S1 Live imaging of glass5.2-RHS expression in wild type. Dorsal view of wild type embryonic head with glass5.2-RHS expression. Images were taken every 2 minutes.
(AVI)

Video S2 Live imaging of glass5.2-RHS expression in *Notch* mutant. Dorsal view of *Notch* mutant embryonic head with glass5.2-RHS expression. Images were taken every 2 minutes.
(AVI)
