# Supplementary figures and images for: Specification of Drosophila Corpora Cardiaca Neuroendocrine Cells from Mesoderm Is Regulated by Notch Signaling
Source: PLoS Genet. 2011 Aug 25;7(8):e1002241. doi: 10.1371/journal.pgen.1002241 (PMC3161926; doi:10.1371/journal.pgen.1002241)

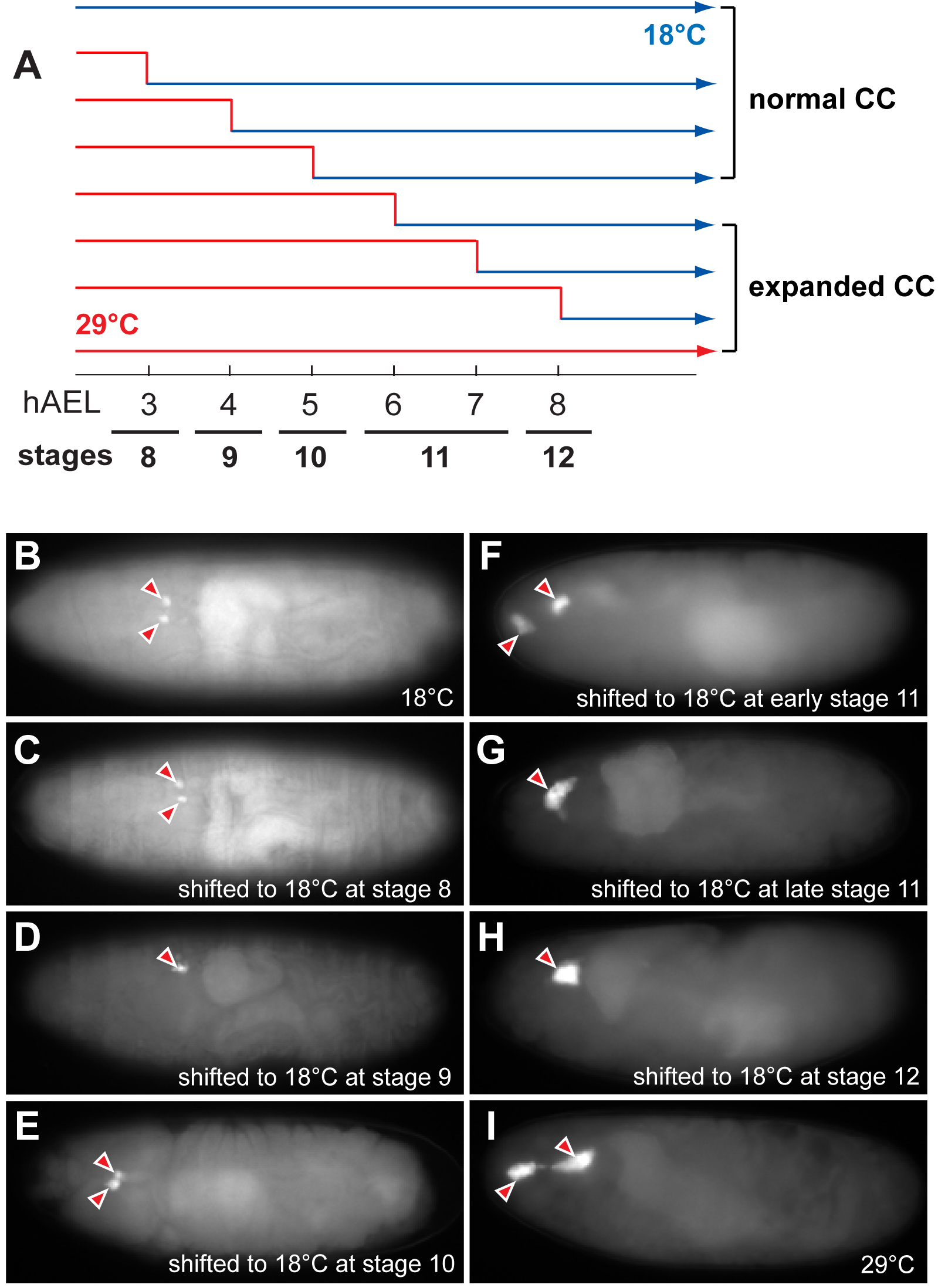

Supplement: Figure S1 — Delta regulates CC cell number before embryonic stage 11. (A) Temperature shift conditions applied to Delta RF mutants at different time points following a 1-hour egg lay. hAEL is ‘hours after egg lay’. 18°C is the permissive temperature, and 29°C is the restrictive temperature for Delta RF mutants. (B) Delta RF mutant grown at 18°C shows normal CC cell appearance, indicated by normal akh-GHP expression (arrowheads). (C–D) Delta RF mutant grown at 29°C for 3 hours (C) or 4 hours (D) followed by a shift to 18°C until stage 17 shows normal CC cell appearance (arrowheads). (E) Delta RF mutant shifted from 29 to 18°C at 5 hAEL showing normal CC cell development, accompanied by an anterior shift of CC cell position. (F) Delta RF mutant with a temperature shift at 6 hAEL shows a modest CC expansion. (G–H) Delta RF mutant with a temperature shift at 7 hAEL (G) or 8 hAEL (H) shows clear CC expansion. (I) Delta RF mutant grown continuously at 29°C exhibits akh-GHP+ CC expansion. All panels show dorsal views of embryos at late stage 17, with anterior to the left. (TIF) [file pgen.1002241.s001.tif]

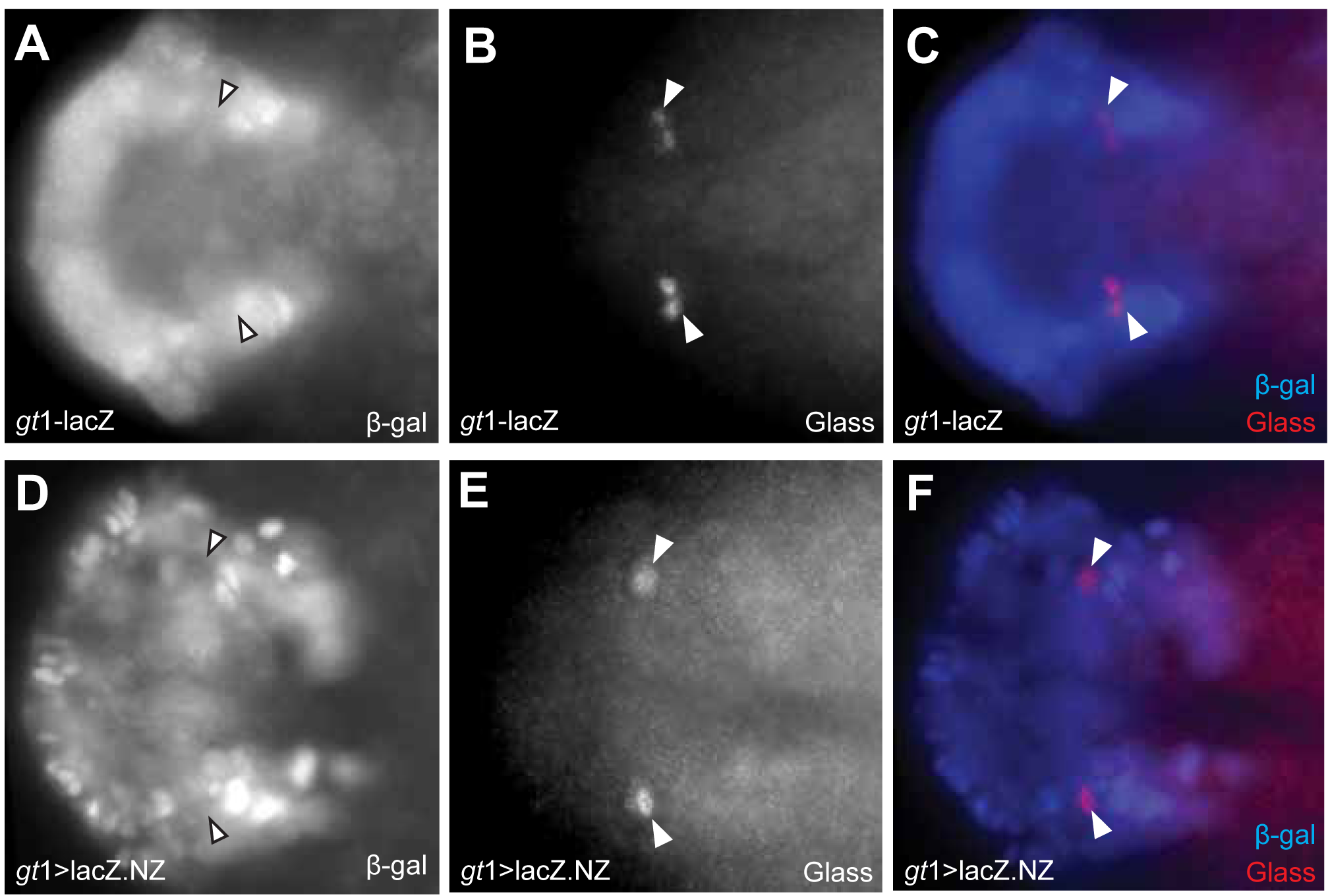

Supplement: Figure S2 — β-gal expression pattern comparison of gt1-lacZ and gt1-GAL4 UAS-lacZ.NZ in anterior head neuroectoderm. (A–C) Expression of β-gal (A) and Glass (B) in the anterior head of stage 11 gt1-lacZ embryo. (D–F) Expression of β-gal (D) and Glass (E) in the anterior head of stage 11 gt1-GAL4 UAS-lacZ.NZ embryo. Arrowheads mark Glass expressing CC precursors (B–C and E–F) and their locations (A and D). (TIF) [file pgen.1002241.s002.tif]

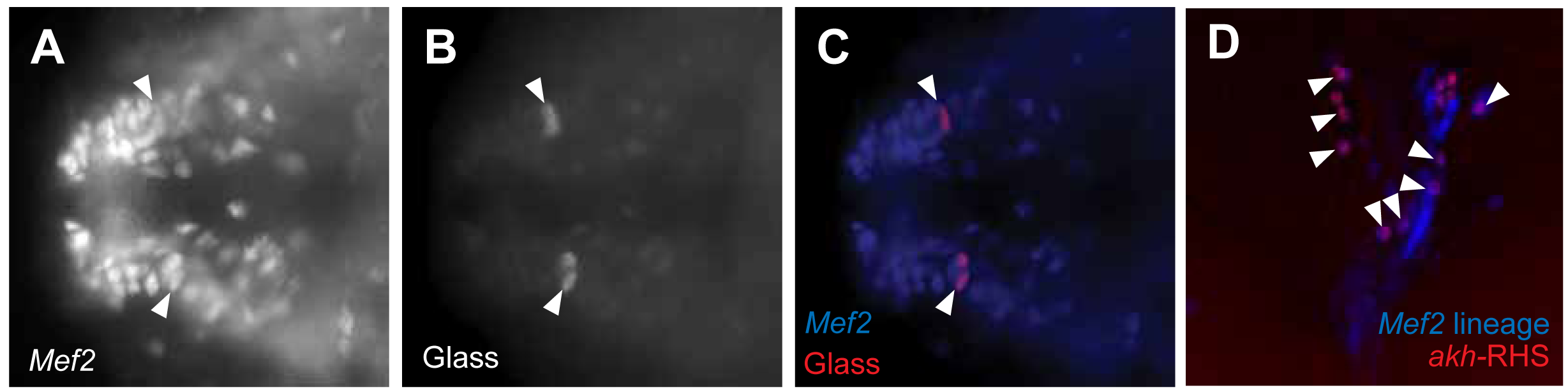

Supplement: Figure S3 — CC cells originate from Mef2-GAL4 expressing mesoderm. (A–C) Glass+ CC precursors in stage 11 embryos are a part of Mef2-GAL4+ cells. The dorsal head mesoderm marked by Mef2-GAL4 expression (A, arrowhead), and Glass+ CC precursors (B, arrowhead) are co-localized (C, magenta cells with arrowheads) with ß-gal+ cells in Mef2-GAL4; UAS-LacZ.NZ embryos. (D) Lineage tracing of Mef2-GAL4+ cells in third instar larval CC cells. Lineage was traced by ß-gal expression (blue) in Mef2-GAL4; UAS-FLP; Act5C(FRT.polyA)lacZ.nls1 larvae. Several CC cells (red) are lineage-traced by Mef2-GAL4 expression (magenta cells with arrowheads). All embryo images are stage 11 dorsal views with anterior to the left. (TIF) [file pgen.1002241.s003.tif]

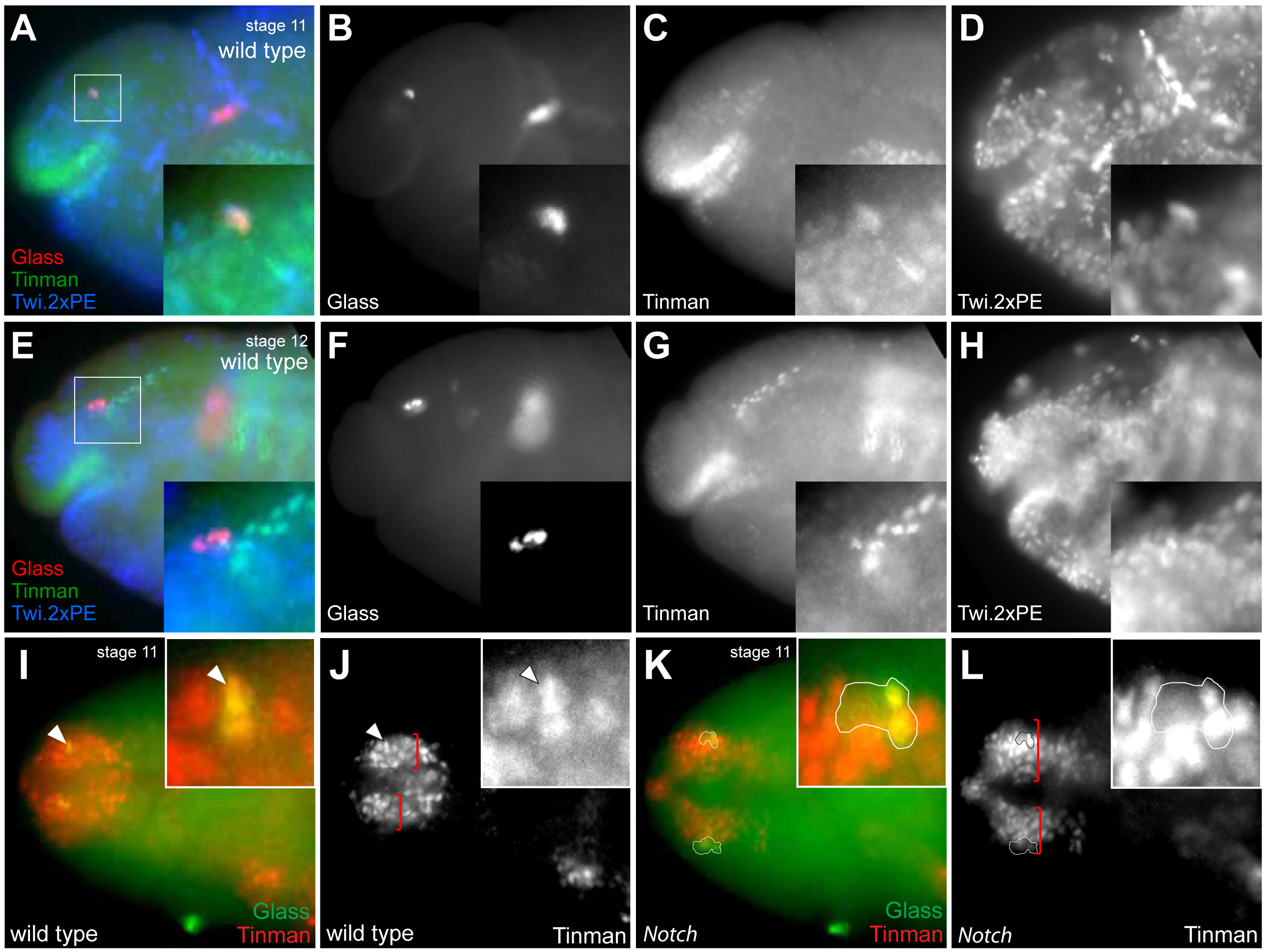

Supplement: Figure S4 — Tinman expression in stage 11 and 12 embryonic head mesoderm. (A–D) Glass+ CC precursors (B) in stage 11 embryos are co-localized with Tinman+ (C) mesodem marked by twi.2×PE-GAL4 UAS-lacZ.NZ (D). The inserts show the enlarged area marked by a box in (A). (E–H) Glass+ CC precursors (F) lose Tinman expression (G), but maintain mesoderm marker (H) shown by twi.2×PE-GAL4 UAS-lacZ.NZ expression. (I, J) Dorsal view of stage 11 wild type embryonic head shows Glass+ CC precursors (arrowhead in I) co-localized with Tinman+ cells (red). The red bracket indicates the width of the Tinman+ cell cluster in wildtype head mesoderm. (K, L) Dorsal view of stage 11 Notch mutant shows multiple Glass+ CC precursors (outlined in insert in K) co-localized with Tinman+ cells (red). The bracket indicates the width of the Tinman+ cell cluster in Notch mutant head mesoderm. All embryo images are lateral views with anterior to the left. (TIF) [file pgen.1002241.s004.tif]
