# Supplementary material for: Specification of Drosophila Corpora Cardiaca Neuroendocrine Cells from Mesoderm Is Regulated by Notch Signaling
Source: PLoS Genet. 2011 Aug 25;7(8):e1002241. doi: 10.1371/journal.pgen.1002241 (PMC3161926; doi:10.1371/journal.pgen.1002241)
Supplement: Table S1 — Identified DrosDel deficiency lines and genetic loci in which mutations resulted in altered number of CC cells. (DOC) [file pgen.1002241.s005.doc]

Table S1. Identified DrosDel deficiency lines and genetic loci in which mutations resulted in altered number of CC cells.

| **Deficiency line** | **Estimated Cytology** | **R5 Coordinates** | **Mapped to loci** | **Alleles tested** |
| --- | --- | --- | --- | --- |
| *Df(1)ED6574* | 2E1;3A2 | X:2078663..2281799 | *crn* | *crn*^yok-EH352^ |
| *Df(1)ED11354* | 2F6;3A4 | X:2219975..2411834 | *gt* | *gt*^X11^ |
| *Df(1)ED6720* | 4B3;4C7 | X:4098384..4438786 | ND |  |
| *Df(1)ED6727* | 4B6;4D5 | X:4219207..4805094 | ND |  |
| *Df(1)ED6906* | 7A3;7B2 | X:7089117..7299839 | ND |  |
| *Df(1)ED6957* | 8B6;8C13 | X:8785828..9029070 | ND |  |
| [*Df(1)ED7147*](http://flybase.org/.bin/fbidq.html?FBab0030759) | 10D7;11A1 | X:11608416..11898833 | ND |  |
| *Df(1)ED7161* | 11A1;11B14 | X:11901120..12644899 | ND |  |
| *Df(1)ED7331* | 13C3;13F1 | X:15344288..15707556 | *sog* | *sog*^U2^ |
| *Df(1)ED7344* | 13E1;13F17 | X:15520480..15762174 | *sog* | *sog*^U2^ |
| *Df(2L)ED1303* | 37E5;38C6 | 2L:19517610..20382385 | *spi* | *spi*^1^ |
| *Df(2L)ED1473* | 39B4;40A5 | 2L:21250892..21828548 | *dimm* | *dimm*^R8^ |
| *Df(2R)ED1673* | 42E1;43D3 | 2R:2873307..3421058 | *so* | *so*^3^ |
| *Df(2R)ED1715* | 43A4;43F1 | 2R:3214456..3804428 | *so* | *so*^3^ |
| *Df(2R)ED2098* | 47A7;47C6 | 2R:6304366..6786711 | ND |  |
| *Df(2R)ED2222* | 47F13;48B6 | 2R:7340485..7552896 | ND |  |
| *Df(2R)ED2247* | 48A3;48D5 | 2R:7487611..7876225 | ND |  |
| *Df(2R)ED2354* | 50E6;51B1 | 2R:10146550..10462062 | *phyl* | *phyl*^2245^ |
| *Df(2R)ED2426* | 51E2;52B1 | 2R:11016313..11498329 | *dup* | *dup*^a1^ |
| *Df(2R)ED2436* | 51F11;52D11 | 2R:11260565..11887804 | *dup* | *dup*^a1^ |
| *Df(2R)ED3610* | 54F1;55C8 | 2R:13738410..14299538 | *thr,Pcl* | *thr*^1^*, Pcl*^11^ |
| *Df(2R)ED3636* | 55B8;55E3 | 2R:14028250..14526773 | *edl* | *edl*^k06602^ |
| *Df(2R)ED3683* | 55C2;56C4 | 2R:14176374..15116496 | *edl* | *edl*^k06602^ |
| *Df(3L)ED4177* | 61C1;61E2 | 3L:319846..1035182 | ND |  |
| *Df(3L)ED4238* | 61C9;62A4 | 3L:738739..1546931 | *rho* | *rho*^7M43^ |
| *Df(3L)ED4710* | 74D1;75B11 | 3L:17480563..18132399 | ND |  |
| *Df(3L)ED4789* | 76A1;76A5 | 3L:19163806..19288762 | ND |  |
| *Df(3R)ED5021* | 81F6;82A5 | 3R:22995..216113 | ND |  |
| *Df(3R)ED5046* | 81F6;82D2 | 3R:22995..564853 | ND |  |
| *Df(3R)ED5071* | 81F6;82E4 | 3R:22995..778404 | ND |  |
| *Df(3R)ED5100* | 81F6;82E7 | 3R:22995..912807 | ND |  |
| *Df(3R)ED5142* | 82B2;82F8 | 3R:279018..1090605 | ND |  |
| *Df(3R)ED5095* | 82C5;82E7 | 3R:475607..912807 | ND |  |
| *Df(3R)ED5138* | 82D5;82F8 | 3R:606794..1090605 | ND |  |
| *Df(3R)ED5644* | 88A4;88C9 | 3R:9843625..10451431 | ND |  |
| *Df(3R)ED5797* | 90C2;90F10 | 3R:13543832..14068391 | *htl* | *htl*^d07110^*, htl*^AB42^ |
| *Df(3R)ED5815* | 90F4;91B8 | 3R:13993596..14484708 | *gl* | *gl*^60j^*, gl*^2^ |
| *Df(3R)ED5942* | 91F12;92B3 | 3R:15052016..15660809 | *Dl* | *Dl*^9P^*, P{EPgy2}EY03425* |
| *Df(3R)ED6076* | 93E10;94A1 | 3R:17459227..17868550 | ND |  |
| *Df(3R)ED6085* | 93F14;94B5 | 3R:17706717..18413461 | ND |  |

ND: Loci attributed to deficiency phenotype were not determined. CC cells are identified by *akh*-RHS reporter.
